# Survey of urinary nickel in peritoneal dialysis patients

**DOI:** 10.18632/oncotarget.19730

**Published:** 2017-07-31

**Authors:** Ya-Ching Huang, Hsiao-Chen Ning, Shang-Syuan Chen, Chia-Ni Lin, I-Kwan Wang, Shu-Man Weng, Cheng-Hao Weng, Ching-Wei Hsu, Wen-Hung Huang, Jang-Jih Lu, Tsu-Lan Wu, Tzung-Hai Yen

**Affiliations:** ^1^ Department of Laboratory Medicine, Chang Gung Memorial Hospital, Linkou, Taiwan; ^2^ Department of Medical Biotechnology and Laboratory Science, College of Medicine, Chang Gung University, Linkou, Taiwan; ^3^ Department of Nephrology, Chang Medical University Hospital and College of Medicine, China Medical University, Taichung, Taiwan; ^4^ Department of Nephrology, Chang Gung Memorial Hospital and College of Medicine, Chang Gung University, Linkou, Taiwan; ^5^ Kidney Research Center, Chang Gung Memorial Hospital, Linkou, Taiwan; ^6^ Center for Tissue Engineering, Chang Gung Memorial Hospital, Linkou, Taiwan

**Keywords:** peritoneal dialysis, nickel, high sensitivity C-reactive protein, inflammation, inductively coupled plasma mass spectrometry

## Abstract

This study surveyed urinary nickel concentrations in peritoneal dialysis (PD) patients, and analyzed the association of urinary nickel concentrations with clinical outcomes and inflammatory biomarkers. In total, 50 PD patients and 50 healthy controls were recruited for this study. All participants were examined for the presence of toxic trace elements (antimony, arsenic, bismuth, cadmium, copper, manganese, mercury, nickel, lead, tellurium, thallium and zinc) in their urine by using inductively coupled plasma mass spectrometry (ICP-MS). It was found that PD patients demonstrated higher urinary nickel concentrations than healthy controls (6.1±3.5 versus 2.8±1.4 μg/L, P<0.001). There were 24 (48.0%) PD patients with normal urinary nickel concentrations, and 26 (52.0%) PD patients with high urinary nickel concentrations. The PD patients with high urinary nickel concentrations demonstrated higher log serum levels of high sensitivity C-reactive protein (0.4±0.5 versus 0.1±0.5 mg/L, P=0.046) than patients with normal urinary nickel concentrations. Furthermore, patients with high urinary nickel concentrations exhibited higher levels of cadmium (1.3±0.9 versus 0.6±0.5 μg/L, P<0.001), copper (7.7±5.7 versus 3.3±1.4 μg/L, P<0.001) and manganese (0.9±1.1 versus 0.4±0.4 μg/L, P=0.023) than patients with normal urinary nickel concentrations. Nevertheless, there were no significant differences in the clinical outcomes between PD patients with high and normal urinary nickel concentrations (P>0.05). Thus, it is concluded that approximately half of the patients undergoing PD had elevated urinary nickel levels, and these patients also had elevated serum levels of high sensitivity C-reactive protein. Nevertheless, no other real correlations were discovered including no impact on patient outcome. Further studies are warranted.

## INTRODUCTION

Accumulation of toxic trace elements in dialysis patients may result from environmental or dietary exposure, or caused by contaminated dialysate [1]. Dialysis patients are at increased danger for excess of toxic trace elements, because they are at exposed to very high titers of dialysate, and so even otherwise minor concentrations gradients of toxic metals between blood and dialysate may lead to considerable systemic toxicities [2]. Furthermore, lack of endogenous renal clearance in dialysis patients may also predispose them to accumulation of the environmental toxic trace elements even not present in dialysate [2]. In a meta-analysis [3], it has been revealed that blood levels of cadmium, chromium, copper, lead, and vanadium were higher and that levels of selenium, zinc and manganese were lower in hemodialysis patients than healthy controls [3].

Iatrogenic nickel intoxication by dialysis has been reported in the literature [4]. The acute nickel poisoning was observed in a group of 23 hemodialysis patients when leaching of nickel-plated stainless steel water heater tank contaminated the dialysate. Symptoms happened during and after dialysis at plasma nickel concentrations of approximately 3 mg/L. Symptoms included nausea, vomiting, weakness, headache, and palpitation. Remission of symptoms occurred at 3 - 13 hours after ending of hemodialysis treatment. The observation indicated that the nickel became bound in the plasma after crossing the membrane, resulting in a higher concentrations in the plasma than in the dialysate and preventing its removal by dialysis [4].

Table 1 highlights the seemingly opposite findings of the various studies of serum nickel level in chronic dialysis patients. Some groups [5–10] reported increased serum nickel concentration in dialysis population. For example, Drazniowsky et al [5] demontrated that median serum nickel concentrations were 1.0 μg/L in healthy controls and 1.6 μg/L in patients with chronic renal failure, respectively. Significantly increased nickel concentrations were found in patients treated by peritoneal dialysis and hemodialysis (8.6 μg/L, P < 0.001). In patients on hemodialysis, post-dialysis nickel concentrations were significantly higher than pre-dialysis values (8.8 μg/L versus 8.6 μg/L, P < 0.001). Previous data from Clinical Laboratory of Chang Gung Memorial Hospital [9] also disclosed that serum nickel concentrations were significantly higher in hemodialysis patients than healthy controls (P < 0.01) and the degree of nickel excess correlated positively with duration of hemodialysis. According to a recent study [8], hemodialysis patients were also found to have significantly higher serum concentrations of nickel than healthy controls (P < 0.0001). On the other hand, some groups [11–16] reported decrease in serum nickel level in dialysis population. In a study by Hosokawa et al [13], serum nickel concentration and creatinine clearance were measured in 20 healthy controls and 40 patients (5 mild renal dysfunction, 10 chronic renal failure, 5 uremia and 20 hemodialysis). The serum nickel levels decreased with the decrement of the creatinine clearance levels. In another subsequent study [15], the relationship between serum nickel concentration and medical complications was examined in 100 hemodialysis patients. The levels of serum nickel were low, and were correlated with total serum protein levels. In another study, Prodanchuk et al [14] also disclosed that significantly decreased serum nickel levels in patients with end-stage renal disease than healthy controls (P = 0.0152). Nevertheless, there was no significant difference in serum nickel levels between hemodialysis and hemodialfiltration (P = 0.832).

**Table 1:** Published studies of nickel exposure in chronic dialysis population

Study	Year	Geographic area	Number of dialysis patients	Type of dialysis	Serum nickel concentration
Drazniowsky et al [5]	1985	United Kingdom		Hemodialysis and peritoneal dialysis	Increased
Hosokawa et al [13]	1988	Japan	20	Hemodialysis	Decreased
Nixon et al [10]	1989	USA	27	Hemodialysis	Increased
Hopfer et al [6]	1989	Canada	30	Hemodialysis	Increased
Hosokawa et al [15]	1990	Japan	100	Hemodialysis	Decreased
Kaminska-Galwa et al [11, 12]	1993-1994	Poland	52	Hemodialysis	Decreased
Hsieh et al [9]	2006	Taiwan	77	Hemodialysis	Increased
Esfahani et al [16]	2007	Iran	40	Hemodialysis	Decreased
Katko et al [7]	2008	Hungary	122	Hemodialysis	Increased
Prodanchuk et al [14]	2014	Ukraine	41	Hemodialysis	Decreased
Gomez de Ona et al [8]	2016	Spain	57	Hemodialysis	Increased
Current study	2017	Taiwan	50	Peritoneal dialysis	Increased (urine)

Therefore, the objective of this study was to evaluate urinary nickel concentrations in PD patients, and to analyze the association of urinary nickel concentrations with clinical outcomes and inflammatory biomarkers.

## RESULTS

**Figure 1:**
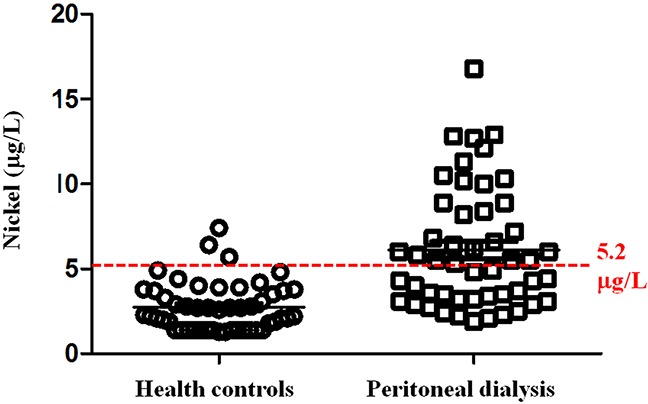
Urinary nickel concentrations Peritoneal dialysis patients demonstrated higher urinary nickel concentrations than healthy controls (6.1 ± 3.5 versus 2.8 ± 1.4 μg/L, P < 0.001). Dashed line is upper reference limit.

Table 2 showed that PD patients were older (51.3 ± 14.0 versus 40.2 ± 9.2 years, P < 0.001) and demonstrated higher serum creatinine levels (12.1 ± 3.7 versus 0.7 ± 0.2 mg/dl, P < 0.001) than healthy controls. The PD patients demonstrated higher urinary nickel concentrations than healthy controls (6.1 ± 3.5 versus 2.8 ± 1.4 μg/L, P < 0.001, Table 2 and Figure 1). Although there were significant differences in urinary excretion of copper, mercury and thallium between PD patients and healthy controls, the urine concentrations of these 3 elements (copper, mercury and thallium) were within normal limits. There were no significant differences in the excretion of other toxic trace elements such as antimony, arsenic, bismuth, cadmium, manganese, lead, tellurium, and zinc (P < 0.05).

**Table 2:** Urinary concentrations of toxic trace element in peritoneal dialysis patients and healthy controls (n = 100)

Variable	All patients (n = 100)	Healthy controls (n = 50)	Peritoneal dialysis patients (n = 50)	P value
Male, n (%)	44 (44%)	20 (40%)	24 (48%)	0.230
Age, years	45.9 ± 13.1	40.2 ± 9.2	51.3 ± 14.0	<0.001***
Nickel workers, n (%)	0 (0)	0 (0)	0 (0)	1.000
Serum creatinine, mg/dL	6.4 ± 6.3	0.7 ± 0.2	12.1 ± 3.7	<0.001***
Urine antimony, μg/L (normal ≤ 7.0)	0.1 ± 0.2	0.1 ± 0.0	0.2 ± 0.2	0.051
Urine arsenic, μg/L (normal ≤ 100.0)	38.3 ± 28.0	43.2 ± 34.4	33.4 ± 18.8	0.080
Urine bismuth, μg/L (normal ≤ 2.0)	0.1 ± 0.0	0.1 ± 0.1	0.1 ± 0.0	0.168
Urine cadmium, μg/L (normal ≤ 2.6)	1.1 ± 0.8	1.2 ± 0.8	1.0 ± 0.8	0.231
Urine copper, μg/L (normal ≤ 80)	34.8 ± 39.7	13.7 ± 7.9	55.8 ± 47.1	<0.001***
Urine lead, μg/L (normal ≤ 23.0)	< 0.6	< 0.6	< 0.6	1.000
Urine manganese, μg/L (normal ≤ 7.9)	0.5 ± 0.7	0.5 ± 0.4	0.6 ± 0.9	0.171
Urine mercury, μg/L (normal ≤ 10.0)	1.0 ± 0.2	1.0 ± 0.3	0.9 ± 0.0	0.035*
Urine nickel, μg/L (normal ≤ 5.2)	4.4 ± 3.2	2.8 ± 1.4	6.1 ± 3.5	<0.001***
Urine tellurium, μg/L (normal ≤ 25.0)	0.1 ± 0.1	0.1 ± 0.1	0.2 ± 0.1	0.566
Urine thallium, μg/L (normal ≤ 10.0)	0.2 ± 0.1	0.2 ± 0.1	0.1 ± 0.0	<0.001***
Urine zinc, μg/L (normal 150 - 1200)	426.8 ± 661.3	445.2 ± 256.6	408.4 ± 904.0	0.782

Note: *P<0.05, ***P<0.001.

As shown in Table 3, there were 24 (48.0%) PD patients with normal urinary nickel concentrations, and 26 (52.0%) PD patients with high urinary nickel concentrations. No significant differences in baseline characteristics between 2 groups (P > 0.05).

**Table 3:** Baseline characteristics of peritoneal dialysis patients, stratified according to urinary nickel concentrations (n = 50)

Variable	All patients (n = 50)	Patients with normal urinary nickel concentrations (n = 24)	Patients with high urinary nickel concentrations (n = 26)	P value
Age, years	51.3 ± 14.0	51.6 ± 14.2	51.1 ± 14.2	0.900
Male, n (%)	24 (48.0)	13 (54.2)	11 (42.3)	0.402
Diabetes mellitus, n (%)	13 (26.0)	4 (16.7)	9 (34.6)	0.148
Hypertension, n (%)	39 (78.0)	18 (75.0)	21 (80.8)	0.623
Coronary heart disease, n (%)	5 (10.0)	1 (4.2)	4 (15.4)	0.187
Hepatitis B surface antigen, n (%)	10 (20.0)	5 (20.8)	5 (19.2)	0.310
Hepatitis C antibody, n (%)	3 (6.0)	1 (4.2)	2 (7.7)	0.511
Smoking habit, n (%)	6 (12.0)	4 (16.7)	2 (7.7)	0.329
Alcohol consumption, n (%)	3 (6.0)	3 (12.5)	0 (0)	0.063
Duration of dialysis, month	34.3 ± 25.5	36.5 ± 26.6	32.5 ± 24.8	0.585

Table 4 shows laboratory findings of PD patients, stratified according to urinary excretion level of nickel. Notably, PD patients with high urinary nickel concentrations demonstrated higher log serum levels of high sensitivity C-reactive protein (0.4 ± 0.5 versus 0.1 ± 0.5 mg/L, P = 0.046) than patients with normal urinary nickel concentrations. There were no significant differences in other parameters (P > 0.05).

**Table 4:** Blood tests of peritoneal dialysis patients, stratified according to urinary nickel concentrations (n = 50)

Variable	All patients (n = 50)	Patients with normal urinary nickel concentrations (n = 24)	Patients with high urinary nickel concentrations (n = 26)	P value
Urine nickel, μg/L	6.1 ± 3.5	3.3 ± 0.9	8.7 ± 3.0	<0.001***
Blood urea nitrogen, mg/dL	73.4 ± 22.0	74.4 ± 18.9	72.4 ± 25.1	0.750
Creatinine, mg/dL	12.1 ± 3.7	12.8 ± 4.2	11.3 ± 3.0	0.155
Uric acid, mg/dL	7.4 ± 1.3	7.6 ± 1.2	7.2 ± 1.5	0.284
Sodium, mEq/L	137.6 ± 3.3	137.1 ± 3.6	138.0 ± 3.0	0.335
Potassium, mEq/L	4.0 ± 0.6	4.2 ± 0.6	3.9 ± 0.6	0.067
Calcium, mg/dL	9.7 ± 0.8	9.8 ± 0.8	9.7 ± 0.8	0.736
Inorganic phosphorus, mg/dL	5.9 ± 1.3	5.7 ± 1.3	6.0 ± 1.3	0.387
Fasting glucose, mg/dL	108.0 ± 43.1	97.4 ± 21.6	116.9 ± 54.0	0.119
Glycated hemoglobin, %	5.9 ± 1.2	5.6 ± 0.5	6.1 ± 1.5	0.119
Albumin, g/dL	3.9 ± 0.4	3.9 ± 0.4	3.9 ± 0.4	0.856
Aspartate transaminase, U/L	25.7 ± 12.8	27.2 ± 13.9	24.3 ± 11.9	0.427
Alanine aminotransferase, U/L	20.0 ± 10.4	19.5 ± 8.7	20.4 ± 11.9	0.747
Alkaline phosphatase, U/L	71.7 ± 28.5	63.5 ± 25.8	78.9 ± 29.3	0.059
Total cholesterol, mg/dL	199.4 ± 47.1	195.0 ± 36.4	203.4 ± 55.3	0.539
High-density lipoprotein, mg/dL	44.9 ± 15.8	48.4 ± 18.0	41.9 ± 13.3	0.153
Low-density lipoprotein, mg/dL	152.5 ± 155.4	152.8 ± 181.9	152.2 ± 132.7	0.989
Triglyceride, mg/dL	185.6 ± 171.9	179.7 ± 193.4	190.5 ± 155.2	0.831
Red blood cell count, 10^6^/μL	3.6 ± 0.5	3.6 ± 0.5	3.6 ± 0.6	0.848
Hemoglobin, g/dL	10.7 ± 1.5	10.8 ± 1.4	10.6 ± 1.6	0.640
Hematocrit, %	31.4 ± 5.3	31.3 ± 6.0	31.5 ± 4.7	0.919
Mean corpuscular volume, fL	88.6 ± 6.2	90.1 ± 4.6	87.2 ± 7.2	0.104
Platelet count, 10^3^/μL	226.0 ± 69.5	211.2 ± 63.2	239.0 ± 73.3	0.163
White blood cell count, 10^3^/μL	7.0 ± 1.9	7.0 ± 1.4	6.9 ± 2.3	0.973
Ferritin, ng/mL	434.7 ± 708.1	570.9 ± 858.0	314.7 ± 533.7	0.220
Iron, μg/dL	83.9 ± 36.1	85.4 ± 27.0	82.6 ± 43.1	0.797
Total iron binding capacity, μg/dL	300.7 ± 61.6	286.9 ± 53.8	312.9 ± 66.4	0.151
Intact parathyroid hormone, pg/mL	374.1 ± 377.3	425.1 ± 388.8	330.9 ± 369.4	0.394
Log (high sensitivity C-reactive protein), mg/L	0.3 ± 0.5	0.1 ± 0.5	0.4 ± 0.5	0.046*

Note: *P<0.05, ***P<0.001.

Table 5 shows association between urinary nickel concentrations with other toxic metals in the PD patients. Patients with high urinary nickel concentrations had higher urinary levels of cadmium (1.3 ± 0.9 versus 0.6 ± 0.5 μg/L, P < 0.001), copper (7.7 ± 5.7 versus 3.3 ± 1.4 μg/L, P < 0.001) and manganese (0.9 ± 1.1 versus 0.4 ± 0.4 μg/L, P = 0.023) than patients with normal urinary nickel concentrations.

**Table 5:** Urinary excretions of other toxic trace elements in peritoneal dialysis patients, stratified according to urinary nickel concentrations (n = 50)

Variable	All patients (n = 50)	Patients with normal urinary nickel concentrations (n = 24)	Patients with high urinary nickel concentrations (n = 26)	P value
Urine antimony, μg/L (normal ≤ 7.0)	0.2 ± 0.2	0.1 ± 0.0	0.2 ± 0.3	0.124
Urine arsenic, μg/L (normal ≤ 100.0)	33.4 ± 18.8	28.6 ± 19.0	37.8 ± 17.8	0.081
Urine bismuth, μg/L (normal ≤ 2.0)	0.1 ± 0.0	0.1 ± 0.0	0.1 ± 0.0	1.000
Urine cadmium, μg/L (normal ≤ 2.6)	1.0 ± 0.8	0.6 ± 0.5	1.3 ± 0.9	0.001**
Urine copper, μg/L (normal ≤ 80)	56 ± 47	33 ± 14	77 ± 57	0.001**
Urine lead, μg/L (normal ≤ 23.0)	0.6 ± 0.0	0.6 ± 0.0	0.6 ± 0.0	1.000
Urine manganese, μg/L (normal ≤ 7.9)	0.6 ± 0.9	0.4 ± 0.4	0.9 ± 1.1	0.023*
Urine mercury, μg/L (normal ≤ 10.0)	0.9 ± 0.0	0.9 ± 0.0	0.0 ± 0.0	1.000
Urine tellurium, μg/L (normal ≤ 25.0)	0.2 ± 0.1	0.2 ± 0.2	0.2 ± 0.1	0.828
Urine thallium, μg/L (normal ≤ 10.0)	0.1 ± 0.0	0.1 ± 0.0	0.1 ± 0.0	1.000
Urine zinc, μg/L (normal 150 - 1200)	408.0 ± 904.0	210.0 ± 494.0	592.0 ± 1142.0	0.137

Note: *P<0.05, **P<0.01.

The relationship among urinary concentrations of nickel, cadmium, copper and manganese are examined using a linear regression model. Simple linear regression analysis indicated that urinary concentrations of cadmium (P < 0.001), manganese (P = 0.002) and copper (P < 0.001) were potential significant determinant of urinary nickel concentration (Table 6). Multiple linear regression analysis revealed that urinary concentrations of cadmium (beta coefficient 2.290, standard error 0.517, P < 0.001) and manganese (beta coefficient 1.329, standard error 0.468, P = 0.007) were independent significant determinants of urinary nickel concentration (Table 6).

**Table 6:** Simple and multiple linear regression analysis for urinary nickel concentration (n=100)

Variable	Simple linear regression		Multiple linear regression		
	R	P value	Beta coefficient	Standard error	P value
Urine cadmium	0.570	<0.001***	2.290	0.517	<0.001***
Urine manganese	0.428	0.002**	1.329	0.468	0.007**
Urine copper	0.559	<0.001***			

Note: **P<0.01, ***P<0.001.

The dialysis related data were summarized in Table 7. There were no significant differences in the dialysis related data such as dialysate/plasma creatinine, peritoneal equilibration test, weekly Kt/V_urea_ and weekly creatinine clearance rate between PD patients with normal or high urinary nickel concentrations (P > 0.05). In addition, none of the 50 PD patients suffered peritoneal dialysis technical failure or died. Finally, there were no significant differences in the total episode of peritonitis between two groups (normal urinary nickel group: 0.4 ± 0.5 versus high urinary nickel group: 0.4 ± 0.9, P = 0.866).

**Table 7:** Dialysis related data of peritoneal dialysis patients, stratified according to urinary nickel concentrations (n = 50)

Variable	All patients (n = 50)	Patients with normal urinary nickel concentrations (n = 24)	Patients with high urinary nickel concentrations (n = 26)	P value
Dialysate/plasma (creatinine)	0.6 ± 0.1	0.6 ± 0.2	0.7 ± 0.1	0.335
Peritoneal equilibration test				0.706
High, n (%)	21 (42.0)	10 (41.7)	11 (42.3)	
High average, n (%)	4 (8.0)	2 (8.3)	2 (7.7)	
Low average, n (%)	21 (42.0)	9 (37.5)	12 (46.2)	
Low, n (%)	4 (8.0)	3 (12.5)	1 (3.8)	
Weekly Kt/V_urea_	2.1 ± 0.4	2.1 ± 0.3	2.1 ± 0.4	0.991
Weekly creatinine clearance rate, L/1.73 m^2^	64.7 ± 16.7	62.8 ± 15.5	66.5 ± 17.8	0.442

## DISCUSSION

In this study, urinary nickel concentrations were used to assess the exposure to nickel, as reported by other groups [17, 18]. The analytical data revealed that approximately half (52.0%) of the patients undergoing PD had elevated urinary nickel levels. Theoretically, PD could lead to a greater depletion of toxic trace elements than hemodialysis because most elements are protein bound and considerable peritoneal protein loss can occur [19]. On the other hand, the volume of dialysate to which the patient is exposed is less for PD than hemodialysis, perhaps reducing the absorption of toxic trace elements [19].

In this study, PD patients were stratified according to their urinary nickel concentrations. Although PD patients also demonstrated higher urinary concentration of copper than healthy controls (55.8 ± 47.1 versus 13.7 ± 7.9 μg/L, P < 0.001), the average urinary concentration of copper was within normal limit. Therefore, copper was not chosen as the index.

Environmental and dietary exposures are considered the main sources of nickel in the general population [20]. Occupational exposure to highly nickel-polluted environments such as nickel refining, electroplating and welding can produce a variety of adverse health effects [21]. Among them are skin allergies, pulmonary fibrosis as well as cancers of the lung, nasal cavity and paranasal sinuses. [21]. The initial effects of nickel toxicity involve irritation of the respiratory tract and nonspecific symptoms. Patients with severe poisoning develop intense pulmonary and gastrointestinal toxicity. Diffuse interstitial pneumonitis and cerebral edema are the principal cause of mortality [20]. Finally, there is also adequate evidence in humans for the carcinogenicity of mixtures that include nickel compounds and nickel metal as documented by International Agency for Research on Cancer, the specialized cancer agency of the World Health Organization [22].

None of our PD patients was nickel workers and there was no occupational contact with nickel. Nickel can enter human body by inhaling nickel-containing air, by drinking nickel-contaminated water, by eating high nickel containing foods, or by wearing nickel-containing jewelry. The government authority has confirmed nickel pollution of certain farm soils in Taiwan [23]. Farm soil has been regarded as an emitter or a receiver of nickel [24]. As an emitter, the soil may release nickel to crops, to the groundwater, or to air. As a receiver, nickel from air or water eventually settles onto the soil [24]. It was reported that fruits and vegetables growing on farm soils with high contents of nickel could contain higher levels of nickel [25]. Previous study [26] also revealed that food is a primary source of nickel exposure for the general population. Common sources of nickel include foods with high nickel content (such as cocoa, chocolate, soya beans, oatmeal, nuts, almonds and fresh and dried legumes, etc.), beverage and dietary supplements with nickel, canned food, nickel-plated utensils as well as stagnated tap water [26]. According to Schaumloffel's study [27], the risk of non-occupational nickel exposure by means of food, drinking water and ambient urban air is low, therefore severe adverse health effects can be excluded. One of the most common harmful health effects is allergic contact dermatitis induced by skin contact of sensitized individuals with nickel [27].

It was shown that PD patients that had elevated urinary nickel levels also had elevated serum levels of high sensitivity C-reactive protein. The C-reactive protein is a biomarker used to measure systemic inflammatory reaction and to predict unfavorable clinical outcomes in PD [28–30]. However, no other real correlations were found in this study including no impact on patient outcome.

Nickel has been found to be the most prevalent allergen in general population, and nickel allergy is a common cause of allergic contact dermatitis [31]. Furthermore, surveys from the North American Contact Dermatitis Group reveal positive patch-test reactions to nickel at around 19.5% of their tested population and a significant increase in the positivity rates for the last decade [32]. Because of the rise in incidents of allergic contact dermatitis to nickel, nickel was voted Allergen of the Year in 2008 by the American Contact Dermatitis Society [33]. Nickel allergy results in both cutaneous and systemic manifestations, and its symptoms may range from mild to severe [34]. A more severe form is systemic nickel allergy syndrome, which is characterized by cutaneous manifestations (systemic contact dermatitis), a chronic course, and systemic symptoms (gastrointestinal, respiratory, neurological, etc.). It is possible that compared with patients with normal urinary nickel concentrations, patients with high urinary nickel concentrations were suffering inflammation (as evidenced by the elevated serum high sensitivity C-reactive protein levels), but poorer outcome was not observed due to relatively short follow up duration.

To avoid the influence of organic arsenic intake, all participants in this study were requested to refrain from ingesting seafood during the 7 days before urine collection. The rational for this practice is, seafood is the largest contributor to organic arsenic exposure in humans and this organic arsenic is considered non-toxic [35]. Urine testing for toxic trace elements is common in the health examinations. High urine total arsenic level due to dietary intake of organic arsenic from seafood may lead to redundant chelation therapy [36]. Therefore, a 7-day abstinence from eating seafood is advised. Otherwise, urinary arsenic speciation is necessary to differentiate between organic and inorganic forms of arsenic.

Nickel is not a cumulative poison, but chronic exposure to nickel may be toxic and carcinogenic. This study has extended this to an association of nickel to chronic dialysis diseases. In summary, approximately half of the patients undergoing PD had elevated urinary nickel levels, and these patients also had elevated serum levels of C-reactive protein. Nevertheless, no other real correlations were discovered in this study including no impact on patient outcome. The limitations of this study include small sample size, lack of nickel blood testing and lack of a hemodialysis control group. Thus, further studies that address these limitations are warranted.

## MATERIALS AND METHODS

### Ethical statement

The study protocol complied with the guidelines of the Declaration of Helsinki and was approved by the Medical Ethics Committee of Chang Gung Memorial Hospital, a *tertiary referral center* located in the northern part of Taiwan. In addition, written informed consent and approval were obtained from all participants before this study.

### Patients

A total of 50 PD patients and 50 healthy controls were recruited between July 2015 and December 2016 from Chang Gung Memorial Hospital, Linkou, Taiwan for this study. All participants were examined for the presence of toxic trace elements (antimony, arsenic, bismuth, cadmium, copper, manganese, mercury, nickel, lead, tellurium, thallium and zinc) in their urine by using ICP-MS. The urinary nickel concentrations were used to assess the exposure to nickel.

### Inclusion and exclusion criteria

Only patients undergoing PD for more than 6 months were enrolled, after excluding those with malignancies, active infectious diseases, hospitalizations or surgery for the past 3 months, and lead [37] or cadmium [38] intoxications.

### PD prescription

The PD prescription for each patient was based on the peritoneal membrane characteristics as determined by the peritoneal equilibration tests, with intermittent therapies used primarily for patients with high transport characteristics and continuous therapies for those with average or low transport characteristics. Low-calcium (1.5 or 1.25 mmol/L), icodextrin-based (7.5 g/dL) or standard dialysates containing glucose (sodium, 135 mmol/L; lactate, 35 mmol/L; calcium, 1.75 mmol/L) were used according to the patients' peritoneal transport characteristics and serum calcium levels to maintain adequate ultrafiltration and normal calcium levels. Dialysis prescription aimed at obtaining a total Kt/V of at least 1.8 per week.

### Laboratory measurements

All laboratory values, including blood cell counts, biochemical data, dialysate/plasma creatinine ratio, peritoneal transport characteristics, weekly creatinine clearance, weekly Kt/V_urea_, were measured by automated and standardized methods. All blood samples were collected in the morning after at least 12 hours of fasting. Serum levels of albumin, blood urea nitrogen, creatinine and transferring saturation were measured and used as nutritional markers. Serum levels of calcium, phosphate, and intact parathyroid hormone were also measured and the corrected serum calcium level was calculated as: calcium (mg/dL) =[0.8 (4.0 - albumin [g/dL])]. All other markers were measured using standard laboratory methods with an automatic analyzer.

### ICP-MS

To avoid the influence of organic arsenic intake, all participants were requested to refrain from ingesting seafood during the 7 days before urine collection. Urine specimens were collected and stored in 10 mL metal-free plastic collection tubes that had been previously decontaminated. To avoid hydration bias during urine sample collection, the urine samples that were over-diluted or over-concentrated (urine creatinine level <10 or >300 mg/dL) were excluded from analysis. Urine specimens were stored at 4°C. A total of 12 toxic trace elements were quantified by means of ICP-MS on a PerkinElmer Elan DRC-e instrument (Waltham, Massachusetts, USA). Antimony, bismuth, cadmium, copper, lead, manganese, nickel, tellurium, thallium and zinc were analyzed using no-gas mode. Methane mode was selected to quantify arsenic, using dynamic reaction cell (DRC) with methane to eliminate polyatomic interferences (for example argon chloride ion). Urine specimens (500 μL) were diluted (1 + 9) with a 1.5% nitric acid (JT Baker, New Jersey, USA) solution containing yttrium as internal standards. Mercury was analyzed independent using no-gas mode. Urine specimens (500 μL) were diluted (1 + 9) with an ethylenediamine tetraacetic acid (0.18%w/v) (Wako, Virginia, USA), cysteine (0.03%w/v) (Sigma, Missouri, USA), hydrochloric acid (15%v/v) (Merck, Darmstadt, Germany), aurum (100 mg/L) solution containing yttrium as internal standards. All the standards were purchased from High-Purity Standards (South Carolina, USA). The following standard ranges were recorded in the instrument software: antimony, bismuth, cadmium, nickel, tellurium and thallium, 1.25 to 40 μg/L; arsenic and copper, 12.5 to 400.0 μg/L; lead, 3.125 to 100.0 μg/L; zinc, 62.5 to 2000 μg/L. Calibration was performed after reagent blank and the six calibration standards in the internal standard diluent solution described above. Calibration curves for all elements had an R ≥ 0.995. Internal quality controls were analyzed at the start and end of each analytical run, and again after every 10 samples. Lypocheck quality controls, urine metals Level 1 and 2 (Bio-Rad Laboratories, Hemel Hempstead, UK) was used for antimony, arsenic, cadmium, copper, lead, manganese, nickel and zinc. In-house prepared quality control was used for the other elements (bismuth, tellurium, thallium and mercury). The lower limit of quantitation (LOQ) for antimony, arsenic, bismuth, cadmium, copper, lead, mercury, manganese, nickel, tellurium, thallium and zinc were 0.1, 1.1, 0.1, 0.3, 3, 0.6, 0.9, 0.1, 1.4, 0.1, 0.1 and 7.6 μg/L, respectively. Values below the LOQ were assigned to LOQ for analysis.

### Statistical analysis

Continuous variables were expressed as a mean with a standard deviation, while categorical variables were expressed as numbers and percentages in brackets. All data were tested for normality of distribution and equality of standard deviation before analysis. As the high sensitivity C-reactive protein data were not normally distributed, these data were log-transformed before analysis. Comparisons between the 2 groups of patients were performed using Student's t test for quantitative variables and Chi-square or Fisher's exact tests for categorical variables. A simple linear regression analysis was performed to compare the frequency of possible risk factors associated with urinary nickel concentration. To control for possible confounding factors, a multiple linear regression analysis (stepwise backward approach) was performed with the factors that were significant in simple linear models (P < 0.05). The criterion for significance was a 95% confidence interval to reject the null hypothesis. All analyses were performed using IBM SPSS Statistics Version 20.0.
